# Global, regional, and national incidence and mortality of neonatal sepsis and other neonatal infections, 1990–2019

**DOI:** 10.3389/fpubh.2023.1139832

**Published:** 2023-03-14

**Authors:** Jie Li, Lin Shen, Kun Qian

**Affiliations:** Department of Reproductive Medicine, Tongji Hospital, Tongji Medical College, Huazhong University of Science and Technology, Wuhan, China

**Keywords:** neonatal sepsis and other neonatal infections, global burden disease, incidence, mortality, global trend

## Abstract

**Background:**

Neonatal infections, especially neonatal sepsis, are one of the major causes of incidence and mortality in pediatrics. However, the global burden of neonatal sepsis and other neonatal infections (NSNIs) remains unclear.

**Methods:**

From the 2019 global disease burden study, we collected annual incident cases, deaths, age-standardized incidence rates (ASIRs), and age-standardized deaths rates (ASDRs) of NSNIs in the past 30 years. Analysis indicators included the percentage of relative changes in incident cases and deaths, and the estimated annual percentage changes (EAPCs) of ASIRs and ASDRs. Correlations were assessed between the EAPCs of ASIRs and ASDRs and social evaluation indicators, including sociodemographic index (SDI) and universal health coverage index (UHCI).

**Results:**

Globally, the number of incident cases of NSNIs grew by 12.79% per year, and the number of deaths dropped by 12.93% per year. During this period, global ASIR of NSNIs increased by 46% annually on average, while ASDR decreased by 53% annually on average. The ASIR and ASDR of female NSNIs were consistently lower than that of male NSNIs. The EAPC of female ASIR was 0.61, nearly twice that of male ASIR, and female ASIR was growing rapidly. The same declining trends of ASDR were noted in males and females. The ASIR of NSNIs in high-SDI regions grew by an average of 14% annually from 1990 to 2019. Except for high-SDI regions, the ASIRs of other 4 SDI regions maintained a rising trend at a high level, and were improved in the past 10 years. The ASDRs of all 5 SDI regions generally showed a downward trend. The region with the highest ASIR of NSNIs was Andean Latin America, and Western Sub-Saharan Africa had the highest mortality. We found a negative correlation between EAPCs of ASDRs and UHCI in 2019.

**Conclusion:**

The global health situation was still not optimal. The incidence of NSNIs remained high, and continues to rise. The mortality of NSNIs has decreased, especially in the countries/territories with high UHCI. Therefore, it is crucial to improve the overall awareness and management of NSNIs, and take interventions for NSNIs worldwide.

## Introduction

Neonatal infections refer to the disease caused by the infection of pathogenic microorganisms (bacterial, viral, or fungal) in newborns, including neonatal sepsis, neonatal pneumonia and other neonatal infections, the most common pathogens of which include group B Streptococcus (GBS), *Escherichia coli*, and herpes simplex virus (HSV) (1, 2). In the past decades, the mechanism of neonatal susceptibility to infection has been extensively studied. Neonatal immature immunity has been implicated for the higher neonatal infection rate (3). Preterm infants, compared with term infants, showed more immature immune responses and had a higher risk of infection (1, 4, 5). Another risk factor was neonatal environmental exposure, such as the maternal history of exposure to infectious diseases and bacterial colonization (6, 7).

Neonatal infections, especially neonatal sepsis, are one of the major causes of incidence and mortality in pediatrics (2). In 2015, 600,000 of newborns died of infection worldwide (8). The 2016 Global Burden of Disease Study illustrated that neonatal sepsis and other neonatal infections (NSNIs) ranked third in neonatal deaths (243,000 deaths per year), and also ranked at the forefront of years of lost life across all age groups (9). It was reported that South Asia and Sub-Saharan Africa had the highest burden of neonatal sepsis, and the total incidence of culture-positive sepsis reported by South Asian hospitals was 15.8 per 1,000 live births (10). This was about 2–4 times higher than the rate reported in the United Kingdom and the United States (1, 11). To reduce the incidence and mortality of NSNIs, measures should be taken based on national and regional incidence and mortality data for NSNIs and related factors.

As far as we know, there is no detailed and systematic study to illustrate the incidence and mortality of NSNIs, and the relationship with national socio-economic status and medical health level. Therefore, we collected Global Burden of Disease 2019 data to assess the global incidence and mortality characteristics of NSNIs and the relationship with national socio-economic status and medical health level, so as to provide a more comprehensive view for the development of global and regional interventions to prevent and control NSNIs health care policies.

## Methods

### Data source

The GBD 2019 study performed an all-round epidemiological assessment of 369 diseases and injuries (by age and sex) in 204 countries and territories worldwide in the past 30 years (12). The details of the GBD study have been reported before (12–14). The specific protocols are available in the GBD website (https://www.healthdata.org/gbd/about/protocol).

From the Global Health Data Exchange (GHDx) query tool, we collected annual incident cases, deaths, age-standardized incidence rates (ASIRs), and age-standardized deaths (ASDRs) of NSNIs in the past 30 years. A total of 204 countries and territories were included, which were divided into 5 sociodemographic index (SDI) regions and 21 GBD regions.

### SDI

The SDI can quantitatively reveal the development status of the country/territory, and it is obtained by comprehensive evaluation of the overall fertility rates of women under the age of 25, the average education level of women aged 15 and above, and the per capita income (15). The SDI values range from 0 to 1. The smaller the SDI, the lower the level of sociodemographic development. The SDIs are provided in the GHDx. The SDIs of 204 countries and territories in 2019 (SDI 2019) are used for subsequent analyses.

### UHCI

Universal health coverage (UHC) refers to the access of all people to the quality health services they need without experiencing financial hardship (16). The UHCI comprises 23 indicators, of which 4 are used to measure intervention coverage, and 19 are based on mortality to measure the quality of care obtained (16). The UHCI is scored on a scale of 0 (less developed) to 100 (most developed). The UHCIs are provided in the GHDx. The UHCIs of 204 countries and territories in 2019 (UHCI 2019) are used for subsequent analyses.

### Statistical analysis

Analysis indicators included the percentage of relative changes in incident cases and deaths, and the estimated annual percentage changes (EAPCs) of ASIRs and ASDRs to characterize the trends in the incidence and mortality of NSNIs. The percentage of relative change in the number of incidents cases and deaths was calculated as reported (17). The formulas for ASIR and ASDR have been described previously (18, 19). EAPC is a measure that reflects trends in the age-standardized rate (ASR) in a certain interval, and its calculation method was described before (20). The EAPC and lower boundary of 95% confidence interval (CI) both >0 indicated an upward trend. Conversely, when both the EAPC and the upper boundary of the 95% CI were lower than 0, ASR showed a downward trend.

In addition, the correlations between the EAPCs of ASIRs and ASDRs and social evaluation indicators (SDI 2019; UHCI 2019) were assessed by Pearson correlation analysis in 204 countries and territories to identify potential factors affecting EAPCs. All analyses were conducted using in R (version 4.1.2). The threshold value of *P* is 0.05.

## Results

### Global trend in NSNIs from 1990 to 2019

**Figure 1:**
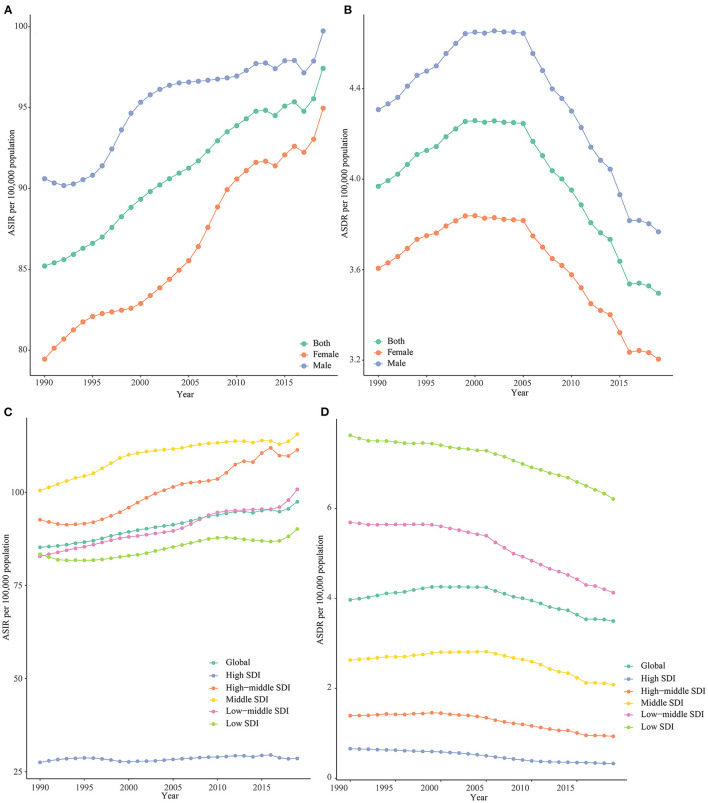
Trends of global ASIRs **(A)** and ASDRs **(B)** by gender and global ASIRs **(C)** and ASDRs **(D)** by SDIs from 1990 to 2019.

On a global scale, the number of incident cases of NSNIs showed a rise of 12.79% (5.59 million in 1990 to 6.31 million in 2019), and the number of deaths of NSNIs declined by 12.93% (260,000 in 1990 to 227,000 in 2019) (Table 1). In males, the number of global incident cases of NSNIs grew by 10.09% annually, and the number of deaths dropped by 12.54% annually. In females, the number of global incident cases of NSNIs grew by 19.52% annually, and the number of deaths dropped by 11.13% annually. After age standardization, the ASIR of NSNIs ascended [EAPC = 0.46 (95% CI, 0.43 to 0.48)] from 85.21 per 100,000 in 1990 to 97.43 per 100,000 in 2019 (Table 2), and the ASDR of NSNIs declined over this period by an average of 0.53 per year (95% CI, −0.72 to −0.35; from 3.97 per 100,000 in 1990 to 3.50 per 100,000 in 2019; Table 2). Overall, the ASIR showed an increasing trend year by year worldwide, with similar trend in males and females, and the ASIR of females was lower than that of males (Figure 1A). It can be noted that the ASIR of male NSNIs (ASIR: 99.74 per 100,000) was higher than that of females (ASIR: 94.96 per 100,000) in 2019 (Table 2). The EAPC of female ASIR was 0.61, nearly twice that of male ASIR, indicating that female ASIR increased rapidly. From 1990 to 2019, the global ASDR initially rose, plateaued between 1999 and 2005, and then declined (Figure 1B). The trend was the same for males and females, with males having higher ASDR than females (Figure 1B).

**Table 1:** Incident cases and deaths of NSNIs in 1990 and 2019 and their change trends from 1990 to 2019.

**Characteristic**	**1990, No**. × **10**^**3**^ **(95% UI)**		**2019, No**. × **10**^**3**^ **(95% UI)**		**Relative change, 1990–2019, %**	
	**Incident cases**	**Deaths**	**Incident cases**	**Deaths**	**Incident cases**	**Deaths**
Overall	5,594.30 (4,004.81–7,551.31)	260.15 (208.54–299.46)	6,310.07 (4,506.66–8,497.41)	226.52 (190.25–275.55)	12.79	−12.93
**Sex**						
Female	2,522.96 (1,810.02–3,421.88)	114.37 (93.63–135.71)	2,970.03 (2,126.63–3,981.09)	100.30 (83.67–120.62)	19.52	−11.13
Male	3,071.33 (2,195.24–4,127.13)	145.78 (113.61–178.68)	3,340.03 (2,388.32–4,473.18)	126.21 (103.06–156.99)	10.09	−12.54
**SDI region**						
Low	854.58 (607.92–1,173.11)	85.50 (65.24–102.06)	1,510.72 (1,066.57–2,075.84)	111.77 (86.93–143.67)	60.36	30.72
Low–middle	1,364.21 (972.90–1,857.99)	102.67 (79.05–124.12)	1,591.86 (1,132.02–2,146.64)	70.06 (57.40–85.91)	11.02	−31.77
Middle	1,932.25 (1,355.95–2,606.77)	54.23 (45.03–60.96)	1,875.71 (1,290.22–2,558.78)	35.82 (29.43–43.14)	−7.66	−33.96
Middle-high	876.24 (604.27–1,204.92)	13.86 (12.29–15.48)	804.06 (555.27–1,087.96)	7.09 (5.91–8.39)	−9.10	−48.81
High	156.42 (121.64–202.45)	3.73 (3.12–4.54)	142.07 (110.56–180.43)	1.63 (1.43–1.86)	−9.17	−56.38
**GBD region**						
Andean Latin America	118.39 (97.19–139.17)	3.67 (2.81–4.74)	110.07 (87.09–130.06)	2.72 (1.88–3.67)	−7.03	−25.90
Australasia	2.12 (1.82–2.80)	0.06 (0.05–0.07)	2.26 (1.99–2.65)	0.02 (0.02–0.03)	3.94	−58.23
Caribbean	49.39 (39.25–59.97)	1.78 (1.40–2.22)	48.83 (39.80–57.52)	1.84 (1.25–2.59)	−1.14	3.66
Central Asia	75.46 (56.83–94.96)	1.05 (0.88–1.25)	63.10 (49.70–78.05)	1.15 (0.91–1.47)	−16.38	9.62
Central Europe	50.66 (37.67–66.71)	0.53 (0.48–0.60)	29.19 (22.17–37.76)	0.12 (0.09–0.15)	−42.38	−78.02
Central Latin America	317.61 (247.84–404.82)	8.47 (7.41–9.53)	339.61 (254.33–432.90)	6.08 (4.67–7.71)	6.93	−28.21
Central Sub-Saharan Africa	76.24 (57.99–101.70)	6.81 (3.84–10.19)	137.80 (102.03–181.84)	9.43 (5.94–14.28)	80.75	38.54
East Asia	1,054.92 (707.11–1,485.15)	4.99 (3.94–5.87)	1,111.97 (747.38–1,544.86)	2.02 (1.69–2.38)	5.41	−59.45
Eastern Europe	213.95 (148.84–289.23)	1.76 (1.58–2.01)	142.19 (96.96–196.71)	1.04 (0.83–1.28)	−33.54	−40.69
Eastern Sub-Saharan Africa	484.38 (345.57–662.44)	36.97 (29.95–44.18)	794.52 (566.60–1,076.46)	46.26 (35.51–60.65)	64.03	25.11
High-income Asia Pacific	14.34 (11.75–17.55)	0.43 (0.34–0.54)	11.00 (9.26–13.09)	0.13 (0.11–0.15)	−23.27	−70.32
High-income North America	55.94 (40.75–77.03)	1.02 (0.94–1.11)	49.57 (36.13–66.66)	0.74 (0.66–0.82)	−11.38	−27.35
North Africa and Middle East	222.98 (159.77–298.87)	10.04 (7.29–12.75)	273.43 (199.95–358.02)	6.71 (5.11–8.69)	22.62	−33.20
Oceania	5.61 (4.14–7.32)	0.19 (0.13–0.28)	9.29 (6.82–12.22)	0.36 (0.21–0.58)	65.54	88.84
South Asia	1,161.08 (824.35–1,606.24)	91.75 (66.71–113.70)	1,428.84 (1,012.92–1,938.41)	55.81 (44.62–69.91)	23.06	−39.17
Southeast Asia	1,016.96 (732.70–1,318.02)	30.95 (23.79–37.98)	745.84 (531.27–997.51)	18.32 (14.43–23.33)	−26.66	−40.81
Southern Latin America	16.06 (13.62–18.58)	1.35 (1.12–1.60)	10.00 (8.25–11.84)	0.55 (0.41–0.73)	−37.75	−58.87
Southern Sub-Saharan Africa	58.87 (41.16–81.27)	3.13 (2.47–3.78)	71.44 (50.05–98.38)	3.43 (2.59–4.48)	21.35	9.68
Tropical Latin America	153.66 (106.36–215.71)	11.67 (10.05–13.69)	168.89 (113.54–238.49)	5.48 (4.27–6.83)	9.91	−53.03
Western Europe	36.57 (31.45–43.44)	0.90 (0.83–0.99)	33.89 (28.59–40.55)	0.44 (0.36–0.53)	−7.33	−50.61
Western Sub-Saharan Africa	409.07 (300.15–560.13)	42.63 (32.77–53.39)	728.34 (541.17–987.94)	63.84 (50.45–81.44)	78.05	49.76

GBD, Global Burden of Disease; SDI, sociodemographic index; UI, uncertainty interval.

**Table 2:** ASIRs and ASDRs of NSNIs in 1990 and 2019 and their change trends from 1990 to 2019.

**Characteristic**	**No. (95% UI)**				**No. (95% CI)**	
	**1990**		**2019**		**1990-2019**	
	**ASIR per 10** ^5^	**ASDR per 10** ^5^	**ASIR per 10** ^5^	**ASDR per 10** ^5^	**EAPC of ASIR**	**EAPC of ASDR**
Overall	85.21 (60.99 to 114.96)	3.97 (3.18 to 4.57)	97.43 (69.58 to 131.21)	3.50 (2.94 to 4.25)	0.46 (0.43 to 0.48)	−0.53 (−0.72 to −0.35)
**Sex**						
Female	79.45 (56.99 to 107.74)	3.61 (2.95 to 4.28)	94.96 (68.00 to 127.27)	3.20 (2.67 to 3.85)	0.61 (0.57 to 0.65)	−0.51 (−0.67 to −0.34)
Male	90.60 (64.75 to 121.71)	4.31 (3.36 to 5.28)	99.74 (71.32 to 133.56)	3.77 (3.08 to 4.69)	0.33 (0.28 to 0.38)	−0.55 (−0.76 to −0.35)
**SDI region**						
Low	83.36 (60.96 to 113.77)	7.63 (5.82 to 9.10)	90.12 (65.40 to 121.37)	6.21 (4.83 to 7.98)	0.31 (0.27 to 0.36)	−0.64 (−0.72 to −0.56)
Low-middle	82.82 (60.44 to 111.40)	5.69 (4.38 to 6.88)	100.76 (73.41 to 133.61)	4.13 (3.38 to 5.06)	0.60 (0.57 to 0.64)	−1.16 (−1.31 to −1.01)
Middle	100.47 (71.52 to 133.99)	2.63 (2.18 to 2.95)	115.57 (81.09 to 156.02)	2.08 (1.71 to 2.50)	0.43 (0.36 to 0.50)	−0.83 (−1.11 to −0.56)
Middle-high	92.61 (64.75 to 126.75)	1.39 (1.23 to 1.55)	111.34 (78.46 to 148.21)	0.93 (0.78 to 1.10)	0.79 (0.73 to 0.84)	−1.59 (−1.85 to −1.33)
High	27.50 (21.39 to 35.59)	0.65 (0.55 to 0.80)	28.54 (22.20 to 36.23)	0.33 (0.29 to 0.37)	0.14 (0.07 to 0.20)	−2.77 (−2.98 to −2.55)
**GBD region**						
Andean Latin America	205.12 (168.34 to 241.17)	6.38 (4.89 to 8.23)	174.90 (138.40 to 206.58)	4.32 (2.97 to 5.82)	−0.52 (−0.36 to −0.68)	−1.24 (−1.38 to −1.10)
Australasia	14.22 (11.92 to 18.33)	0.37 (0.31 to 0.44)	12.72 (11.24 to 14.95)	0.13 (0.10 to 0.17)	−0.25 (−0.32 to −0.18)	−3.91 (−4.16 to −3.66)
Caribbean	49.39 (39.25 to 59.97)	1.78 (1.40 to 2.22)	48.83 (39.80 to 57.52)	1.84 (1.25 to 2.59)	0.27 (0.05 to 0.48)	0.55 (0.36 to 0.74)
Central Asia	80.66 (60.74 to 101.50)	1.12 (0.94 to 1.34)	69.61 (54.83 to 86.09)	1.27 (1.00 to 1.61)	−0.60 (−0.72 to −0.47)	0.92 (0.66 to 1.18)
Central Europe	62.90 (46.79 to 82.78)	0.65 (0.59 to 0.74)	56.35 (42.82 to 72.86)	0.22 (0.17 to 0.29)	−0.85 (−1.26 to −0.44)	−4.58 (−5.03 to −4.13)
Central Latin America	133.78 (104.38 to 170.51)	3.57 (3.12 to 4.02)	160.76 (120.44 to 204.80)	2.87 (2.20 to 3.64)	0.69 (0.45 to 0.93)	−0.82 (−1.03 to 0.62)
Central Sub-Saharan Africa	59.88 (45.54 to 79.85)	5.36 (3.03 to 8.02)	64.77 (47.96 to 85.51)	4.44 (2.79 to 6.71)	0.30 (0.26 to 0.33)	−0.44 (−0.58 to −0.29)
East Asia	87.64 (58.76 to 123.37)	0.41 (0.33 to 0.49)	149.72 (100.71 to 207.87)	0.27 (0.23 to 0.32)	2.16 (2.04 to 2.27)	−1.79 (−1.96 to −1.63)
Eastern Europe	152.01 (105.76 to 205.43)	1.24 (1.12 to 1.42)	130.81 (89.24 to 180.99)	0.95 (0.76 to 1.17)	−0.64 (−0.70 to −0.58)	−1.37 (−1.70 to −1.04)
Eastern Sub-Saharan Africa	114.12 (81.38 to 156.03)	8.77 (7.10 to 10.48)	117.92 (84.07 to 159.74)	6.88 (5.28 to 9.02)	0.18 (0.09 to 0.28)	−0.64 (−0.79 to −0.49)
High-income Asia Pacific	15.11 (12.38 to 18.49)	0.45 (0.35 to 0.57)	16.53 (13.92 to 19.66)	0.19 (0.16 to 0.22)	−0.08 (−0.02 to 0.04)	−3.69 (−4.00 to −3.39)
High-income North America	25.38 (18.49 to 34.98)	0.46 (0.43 to 0.50)	24.51 (17.87 to 32.93)	0.37 (0.33 to 0.40)	−0.11 (−0.20 to −0.01)	−0.81 (−1.05 to −0.57)
North Africa and Middle East	39.87 (28.57 to 53.46)	1.80 (1.31 to 2.29)	46.94 (34.33 to 61.47)	1.15 (0.87 to 1.49)	0.45 (0.39 to 0.51)	−1.66 (−1.73 to −1.60)
Oceania	52.76 (38.89 to 68.78)	1.79 (1.19 to 2.62)	47.19 (34.63 to 62.10)	1.83 (1.06 to 2.93)	−0.59 (−0.72 to −0.45)	0.17 (−0.02 to 0.36)
South Asia	67.82 (48.15 to 93.74)	5.37 (3.91 to 6.66)	89.21 (63.24 to 121.00)	3.49 (2.79 to 4.37)	0.77 (0.71 to 0.84)	−1.67 (−1.83 to −1.52)
Southeast Asia	168.66 (121.51 to 218.56)	5.14 (3.95 to 6.31)	142.57 (101.51 to 190.57)	3.49 (2.75 to 4.45)	−0.60 (−0.67 to −0.52)	−1.21 (−1.36 to −1.07)
Southern Latin America	31.99 (27.13 to 37.02)	2.68 (2.23 to 3.19)	21.50 (17.75 to 25.45)	1.19 (0.89 to 1.57)	−1.71 (−1.85 to −1.57)	−3.21 (−3.44 to −2.99)
Southern Sub-Saharan Africa	80.03 (55.93 to 110.49)	4.26 (3.36 to 5.14)	89.79 (62.91 to 123.63)	4.31 (3.25 to 5.63)	0.65 (0.45 to 0.84)	0.55 (0.25 to 0.85)
Tropical Latin America	89.89 (62.21 to 126.18)	6.84 (5.89 to 8.02)	109.16 (73.41 to 154.10)	3.53 (2.75 to 4.40)	0.76 (0.65 to 0.88)	−2.30 (−2.63 to −1.97)
Western Europe	16.37 (14.08 to 19.44)	0.40 (0.37 to 0.44)	16.31 (13.76 to 19.51)	0.21 (0.17 to 0.25)	−0.05 (−0.11 to 0.01)	−2.01 (−2.13 to −1.89)
Western Sub-Saharan Africa	95.84 (70.29 to 131.15)	10.05 (7.72 to 12.57)	93.05 (69.12 to 126.21)	8.18 (6.46 to 10.43)	−0.23 (−0.27 to −0.18)	−0.72 (−0.76 to −0.68)

ASIR, age-standardized incidence rate; ASDR, age-standardized deaths rate; EAPC, estimated annual percentage change; GBD, Global Burden of Disease; SDI, sociodemographic index; UI, uncertainty interval.

### Regional trend in NSNIs from 1990 to 2019

In high-SDI regions, the number of incident cases of NSNIs decreased by 9.17%, and the number of deaths of NSNIs decreased by 56.38% from 1990 to 2019 (Table 1). The ASIR of NSNIs during this period increased [EAPC = 0.14 (95% CI, 0.07 to 0.20)] from 27.50 per 100,000 in 1990 to 28.54 per 100,000 in 2019, and the ASDR declined [EAPC = −2.77 (95% CI, −2.98 to −2.55)] from 0.65 per 100,000 in 1990 to 0.33 per 100,000 in 2019 (Table 2). The ASIRs of other 4 SDI regions all maintained a rising trend at a high level except for high-SDI regions, and there were heterogeneous trends between ASIRs and SDIs, for example, the ASIR of middle-SDI regions was not lower than that of low-SDI regions (Table 2, Figure 1C, Supplementary Figure 1A). The ASDR in all 5 SDI regions showed a downward trend on the whole, and the higher the SDI, the smaller the ASDR (Table 2, Figure 1D, Supplementary Figure 1B).

**Figure 2:**
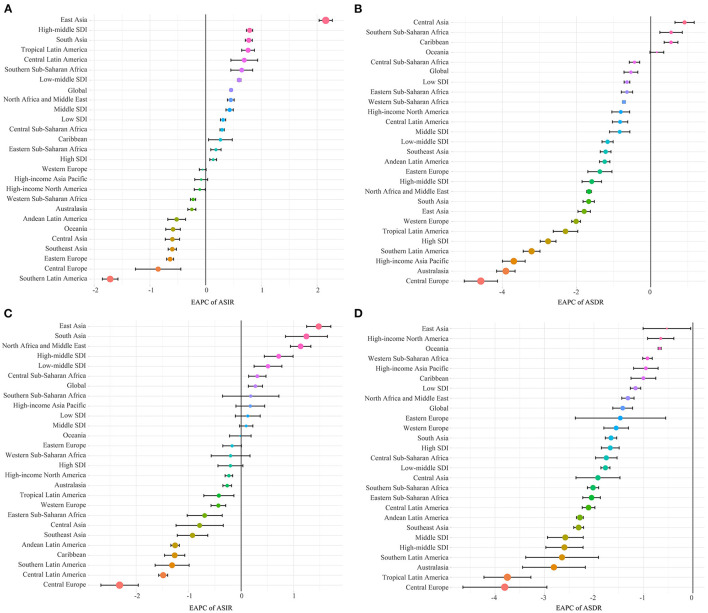
Estimated annual percentage changes (EAPCs) of ASIRs and ASDRs in NSNIs at the regional levels. **(A)** EAPCs of ASIRs from 1990 to 2019; **(B)** EAPCs of ASDRs from 1990 to 2019; **(C)** EAPCs of ASIRs from 2010 to 2019; **(D)** EAPCs of ASDRs from 2010 to 2019.

Among the 21 GBD regions, the number of incident cases increased in 10 regions and decreased in 11 regions; the number of deaths increased in seven regions and decreased in 14 regions (Table 1). The regions where both incident cases and deaths were increasing were Southern Sub-Saharan Africa, Eastern Sub-Saharan Africa, Western Sub-Saharan Africa, Central Sub-Saharan Africa, and Oceania (Table 1). The largest increase in incident cases was in Central Sub-Saharan Africa (relative change = 80.75%), and in deaths in Oceania (relative change = 88.84%; Table 1). Besides, the number of incident cases (relative change = −42.38%) presented the most rapid decline in Central Europe, as did the number of deaths (relative change = −78.02%; Table 1). The region with the highest incidence of NSNIs was Andean Latin America (2019 ASIR: 174.90 per 100,000), and Central Latin America ranked second (2019 ASIR: 160.76 per 100,000) (Table 2). In terms of NSNIs mortality, the threat was most severe in Western Sub-Saharan Africa (2019 ASDR: 8.18 per 100,000), and Eastern Sub-Saharan Africa ranked second (2019 ASDR: 6.88 per 100,000; Table 2). The ASIRs decreased in 10 regions, most notably in Southern Latin America [EAPC = −1.71 (95% CI −1.85 to −1.57)]; the ASIRs of 9 regions had an increasing trend, with East Asia showing highest increasing trend [EAPC = 2.16 (95% CI 2.03 to 2.27); Table 2, Figure 2A]. The ASIRs of Western Europe and High-income Asia Pacific remained stable (Table 2, Figure 2A). The ASDR generally showed a downward trend, with only three regions showing an upward trend, including Central Asia, Southern Sub-Saharan Africa, and the Caribbean (Table 2, Figure 2B). The largest decrease in ASDR was noted in Central Europe (Table 2, Figure 2B).

In order to show the recent changes in the ASIRs and ASDRs of NSNIs, we obtained data from the past 10 years (2010–2019; Supplementary Table 1). In recent 10 years, the ASIRs in regions with high-middle and low-middle SDI were still on the rise, while those in regions with high SDI, middle SDI and low SDI tended to be stable and improved compared with the overall trend (the ASIRs of all SDI regions were on the rise from 1990 to 2019; Figures 2A, C). The short-term and long-term trends of ASDRs in the 5 SDI regions were the same, showing a downward trend (Figures 2B, D). In the 21 GBD regions, East and South Asia remained the top 2 in the rapid growth of ASIRs, while Central Europe and Southern Latin America maintained a continuous decline of ASIRs (Figure 2C). Central Latin America, Eastern Sub-Saharan Africa, Tropical Latin America and Caribbean performed well. While the overall ASIRs (1990–2019) showed an upward trend, effective measures were taken in the past 10 years to successfully curb the upward trend of ASIRs, showing a downward trend (Figures 2A, C). In Southern Sub-Saharan Africa, ASIR stopped growing and remained stable over the past decade (Figure 2C). The ASDRs declined in all 21 GBD regions from 2010 to 2019, indicating that infant death caused by NSNIs has attracted attention in some regions where ASDR was still growing (Figure 2D).

### National trend in NSNIs from 1990 to 2019

Among 204 countries and territories, China (1.11 million) and India (0.75 million) ranked the top 2 in the number of incident cases of NSNIs in 2019, accounting for about 29% of the number of global incident cases (6.31 million; Supplementary Table 2). The countries with the highest number of deaths were India (36,900) and Nigeria (27,200), accounting for 28% of the number of global deaths (226,500; Supplementary Table 2).

**Figure 3:**
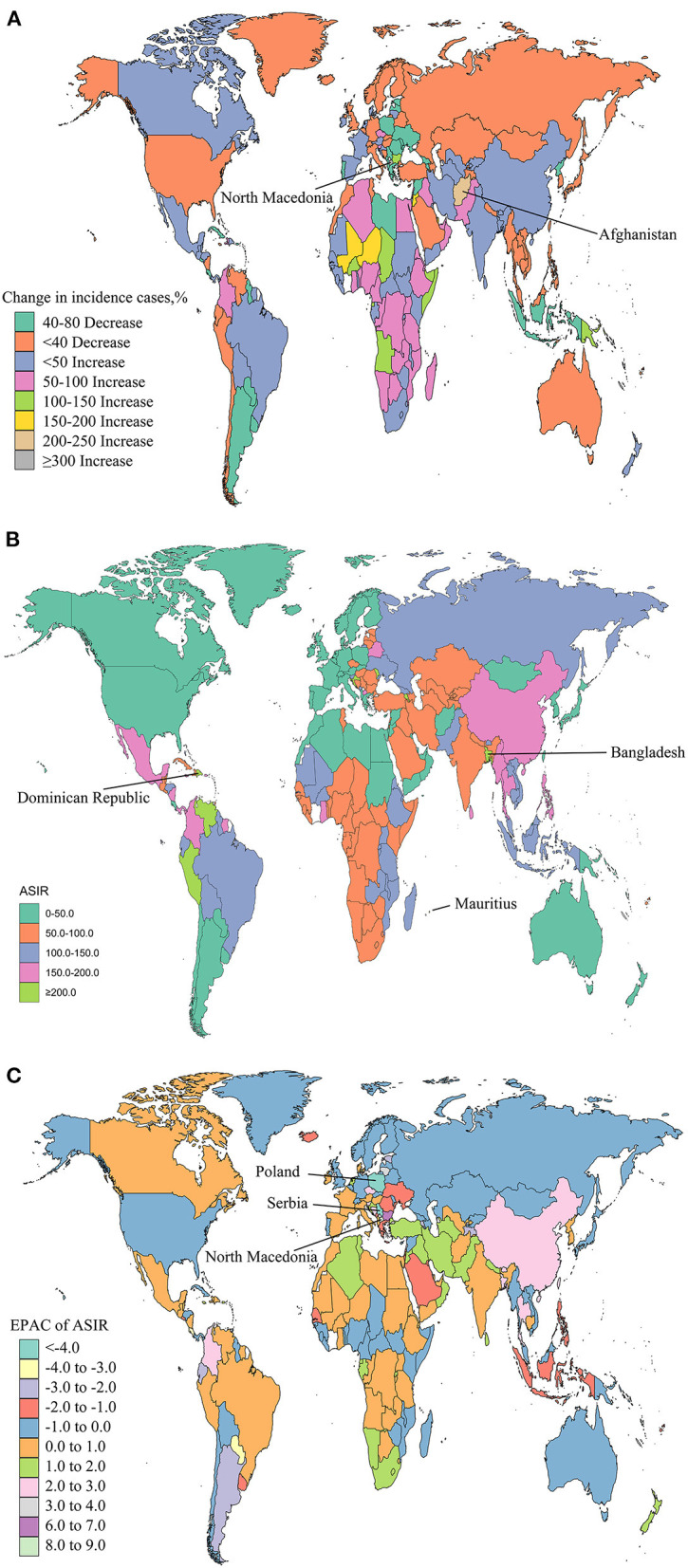
Global trends in the incidence of NSNIs in 204 countries and territories. The percentage of relative change in incident cases of NSNIs between 1990 and 2019 **(A)**, age-standardized incidence rates (ASIRs) of NSNIs in 2019 **(B)**, and estimated annual percentage changes (EAPCs) of ASIRs of NSNIs from 1990 to 2019 **(C)** were shown.

Northern Macedonia exhibited the largest increase in incident cases of NSNIs (300.45%), followed by Afghanistan (241.37%; Supplementary Table 3, Figure 3A). Mauritius had the largest ASIR (244.26 per 100,000), followed by Bangladesh (231.66 per 100,000), and Dominican Republic (230.71 per 100,000; Supplementary Table 2, Figure 3B). The ASIRs of 101 countries or territories showed an upward trend, of which North Macedonia had the largest increase [EAPC = 8.41 (95% CI, 7.72 to 9.10); Supplementary Table 4, Figure 3C]. The ASIRs of 93 countries or territories were considered to show a downward trend, with the largest decline in Serbia [EAPC = −4.76 (95% CI, −5.25 to −4.26)], followed by Poland [EAPC = −4.47 [95% CI, −5.36 to −3.58); Supplementary Table 4, Figure 3C]. The ASIRs of 25 countries or territories, including Denmark, Norway, and Greenland, remained stable (Supplementary Table 4).

**Figure 4:**
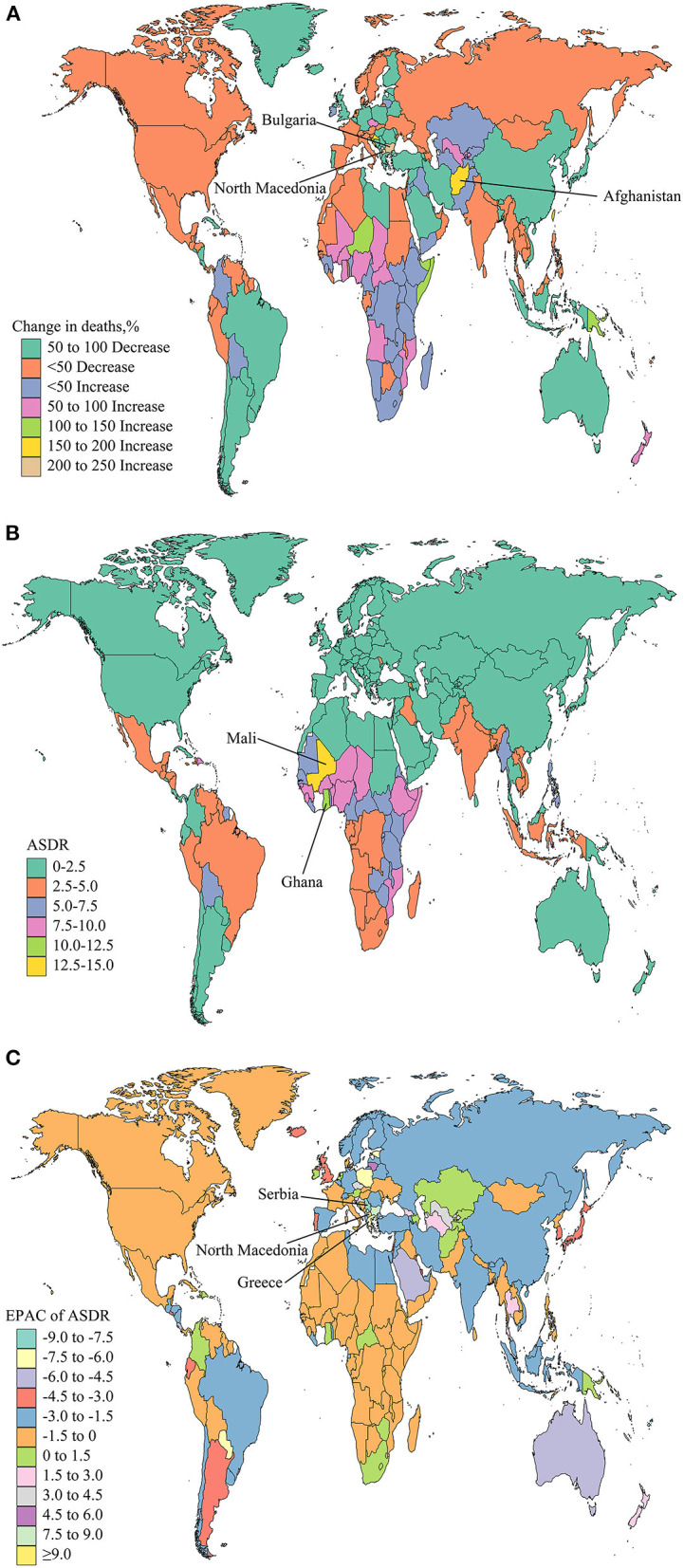
Global trends in the deaths of NSNIs in 204 countries and territories. The percentage of relative change in deaths of NSNIs between 1990 and 2019 **(A)**, age-standardized deaths rates (ASDRs) of NSNIs in 2019 **(B)**, and estimated annual percentage changes (EAPCs) of ASDRs of NSNIs from 1990 to 2019 **(C)** were shown.

North Macedonia (227.49%) had the largest increase in deaths of NSNIs, followed by Bulgaria (216.90%), and Afghanistan (196.38%; Supplementary Table 3, Figure 4A). In 2019, Mali (ASDR: 14.11 per 100,000) and Ghana (ASDR: 10.14 per 100,000) showed the highest ASDR (Supplementary Table 2, Figure 4B). The ASDRs were on the rise among the 38 countries or territories, with the largest increase in North Macedonia [EAPC = 9.31 (95% CI, 7.81 to −10.83); Supplementary Table 4, Figure 4C]. The ASDRs of 153 countries or territories declined, with the greatest reduction in Serbia [EAPC = −8.66 (95%CI, −9.83 to −7.47)], followed by Greece [EAPC = −7.41 (95% CI, −9.32 to −5.46); Supplementary Table 4, Figure 4C]. The ASDR remained stable in 13 countries or territories, including Yemen, Somalia, and Belize (Supplementary Table 4).

To show the effect of recent interventions, we analyzed the changes of ASIRs and ASDRs in 204 countries and territories from 2010 to 2019. The ASIRs of 74 countries or territories showed an upward trend, with the largest increase in Singapore [EAPC = 4.08 (95% CI, 3.92 to 4.24); Supplementary Table 4, Supplementary Figure 2A]. The ASIRs of 92 countries or territories were in a downward trend, with the largest decline in Estonia [EAPC = −6.21 (95% CI, −7.09 to −5.33)], followed by Serbia [EAPC = −5.22 (95% CI, −6.14 to −4.29)] and Poland [EAPC = −5.03 (95% CI, −6.32 to −3.73); Supplementary Table 4, Supplementary Figure 2A]. The ASIRs of 38 countries or territories remained stable, such as Canada and Finland (Supplementary Table 4). The ASDRs of eight countries or territories had a downward trend, and Mauritius had the largest increase [EAPC = 2.98 (95% CI, 1.23 to 4.77)], followed by Austria, Greenland and Singapore (Supplementary Table 4, Supplementary Figure 2B). The ASDRs of 177 countries or territories decreased, of which Estonia experienced the largest decline [EAPC = −7.70 (95% CI, −11.61 to −3.62)], followed by Cyrus [EAPC = −7.22 (95% CI, −8.75 to −5.67); Supplementary Table 4, Supplementary Figure 2B]. The ASDRs remained stable in 19 countries or territories, such as Sweden, France and Greece (Supplementary Table 4). Compared with the data of the past 30 years, the ASIRs and ASDRs of NSNIs in some countries and territories were under control from 2010 to 2019.

### Correlations of EAPCs of ASIR and ASDR with social evaluation indicators

**Figure 5:**
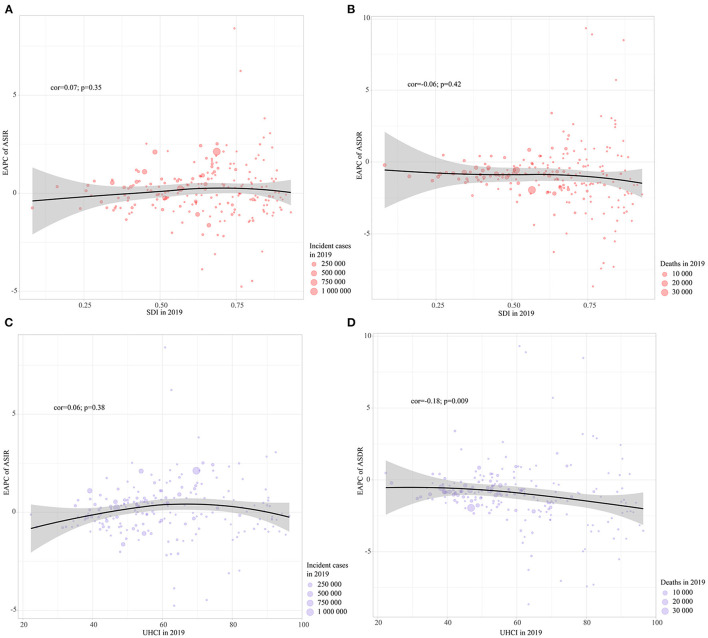
Pearson correlation was analyzed between EAPCs of ASIRs and ASDRs in NSNIs from 1990 to 2019 and SDI and UHCI in 2019 at the country and territorial levels. **(A)** SDI in 2019 and EAPC of ASIR; **(B)** SDI in 2019 and EAPC of ASDR; **(C)** UHCI in 2019 and EAPC of ASIR; **(D)** UHCI in 2019 and EAPC of ASDR. The incident cases and deaths of NSNIs from 204 countries and territories in 2019 are represented by circles. The size of the circles increased with the incident cases or deaths of NSNIs. Correlation coefficient, cor.

The EAPCs of ASIRs and ASDRs of NSNIs in 2019 were not correlated with SDI (*p* > 0.05; Figures 5A, B). The EAPCs of ASIRs of NSNIs in 2019 were not correlated with UHCI (*P* > 0.05; Figure 5C), while the EAPCs of ASDRs of NSNIs in 2019 was significantly negatively correlated with UHCI (cor = −0.18; *P* = 0.009; Figure 5D).

## Discussion

To the best of our knowledge, we for the first time comprehensively assessed global and regional long-term and short-term characteristics in incidence and mortality of NSNIs. In this study, we found that the number of incident cases of NSNIs in the world grew by 12.79% per year, and the number of deaths dropped by 12.93% per year from 1990 to 2019. During this period, global ASIR of NSNIs increased by 46% annually on average, while ASDR decreased by 53% annually on average. The ASIR and ASDR of female NSNIs were consistently lower than that of male NSNIs. The EAPC of female ASIR was 0.61, nearly twice that of male ASIR, and female ASIR was growing rapidly. The same declining trends of ASDR were noted in males and females. For SDI regions, the ASIR of NSNIs in high-SDI regions grew by an average of 14% annually from 1990 to 2019. Except for high-SDI regions, the ASIRs in other 4 SDI regions maintained a rising trend at a high level. The ASDRs of all 5 SDI regions generally showed a downward trend. The largest decline was in the high-SDI regions. The highest ASIR of NSNIs was in Andean Latin America (ASIR in 2019: 174.90 per 100,000), and the highest mortality of NSNIs was in Western Sub-Saharan Africa (ASDR in 2019: 8.18 per 100,000). From 2010 to 2019, the burden of NSNIs in several regions where the ASIRs or ASDRs were rising were under control. We found a negative correlation between EAPCs of ASDRs and UHCI in 2019.

NSNIs are one of the major causes of 2.6 million newborn deaths worldwide every year (21). It is necessary and significant to improve the current situation of NSNIs. We need to better understand the characteristics of NSNIs, and actively enhance newborn care. In this study, we found that the global incidence of NSNIs showed rapid growth, and the mortality was generally declining. The incidence and mortality of males and females were in line with global trends. The incidence and mortality of NSNIs in females were lower than in males. The continuous increase in global incidence of NSNIs may indicate that the global health environment still needs to be improved. However, the decline of global mortality of NSNIs in the short term showed that the current maternal and newborn health care has achieved positive results (22).

We found that in high SDI regions, the ASIR showed the lowest increase, while the ASDR exhibited the highest decline. In other 4 SDI regions, we found no relationship between ASIR and SDI levels. For example, the ASIR in the middle-SDI regions was higher than that in the low-SDI regions. The ASDRs of all 5 SDI regions were generally decreasing, and the higher the SDI, the smaller the ASDR. This finding indicated that NSNIs had a high ASIRs and was growing rapidly, and the mortality of NSNIs was related to the educational level and economic level of the SDI regions. Although the ASIRs of Western Sub-Saharan Africa and Eastern Sub-Saharan Africa were not the highest (the highest was Andean Latin America and Central Latin America), the ASDRs were the highest. Consistent with our findings, Sub-Saharan Africa topped the list for the mortality of NSNIs (23). One possible reason was the high antimicrobial resistance of pathogens in Sub-Saharan Africa, and the efficacy of treatment was reduced (24). Ranjeva et al. evaluated the public health burden and economic pressure caused by neonatal sepsis in Sub-Saharan Africa, and believed that it was necessary to provide reasonable and appropriate health care for neonatal infections (25).

The high incidence and its high growth rate of NSNIs may indicate that infants are generally susceptible to pathogens and their infection capacity is increasing. The death of NSNIs may be related to the lack of timely diagnosis and treatment, and antibiotic resistance (26, 27). Our findings illustrated that the mortality of NSNIs was relatively high in low- and middle-income countries. Families and health workers in low- and middle-income countries lack relevant knowledge and training to identify the early stage of infections, and the corresponding laboratory equipment is only available in hospitals, delaying the time of treatment (27, 28). In addition, many people in low- and middle-income countries may not go to hospitals due to financial hardship, or may not have access to medical care. It was estimated that there were 8 million curable deaths in 2015, of which 96% occurred in low- and middle-income countries (29). A study estimated the quality of care in 81 low- and middle-income countries, and when high-quality care was provided, it was believed that neonatal mortality would be reduced by 28% (30). UHCI is an indicator reflecting health coverage and nursing quality (16). Efforts to improve UHC can overcome these issues and promote health equity. From our results, it was observed that the UHCI was negatively correlated with the trend of ASDRs, that is, when the level of UHCI was high, the ASDR was generally in a downward trend. This was consistent with previous report (31). There is no doubt that the use of antibiotics is crucial to the treatment of infections. It is known that there is a clear correlation between antibiotic use and antibiotic resistance, and antibiotic resistance has become a persistent global health threat (32, 33). On the one hand, there is the misuse of antibiotics and population-level resistance in low- and middle-income countries (34). On the other hand, low- and middle-income countries showed high resistance to antibiotics recommended by World Health Organization (WHO) (35). These are not conducive to neonatal outcomes. Therefore, we need to improve the overall awareness, timely diagnosis and management of NSNIs.

Finally, special attention should be paid to countries or territories where ASDR is still growing, including Mauritius, Austria, and Greenland (Supplementary Table 4). In these countries and territories with ASDR on the rise, it was imperative to accelerate the promotion of universal health coverage and strengthen antibiotic management.

### Limitations

This study fills a gap in this area by providing the first comprehensive assessment of global and regional long-term and short-term characteristics of incidence and mortality of NSNIs, and the relationship with socioeconomic status and medical health level. However, we should recognize several limitations. First of all, the quality of the data limited our accurate description of the results. The possible bias made the results less robust. Second, the causes of NSNIs were notoriously complex. There was no specific pathogen infection data in the GBD study, so we could not estimate the contribution of different causes.

## Conclusions

To sum up, the ASIR of global NSNIs gradually increased and the ASDR gradually declined from 1990 to 2019. In addition, the ASIR and ASDR of males were higher than those of females. Except for high-SDI regions, the ASIRs of other 4 SDI regions remained at a high level in this period. The ASDRs declined in all SDI regions. The ASDRs in Western Sub-Saharan Africa and Eastern Sub-Saharan Africa were the highest. The ASDRs were still growing in some countries and territories. Therefore, it is essential to improve the overall awareness and management of NSNIs, and reduce the morbidity and mortality of NSNIs worldwide.

## Data availability statement

The original contributions presented in the study are included in the article/Supplementary material, further inquiries can be directed to the corresponding author.

## Author contributions

KQ: project design. JL: data curation, analysis, and manuscript writing. LS: manuscript revision. All authors read and approved the final manuscript.
