# Supplementary material for: Global, regional, and national incidence and mortality of neonatal sepsis and other neonatal infections, 1990–2019
Source: Front Public Health. 2023 Mar 14;11:1139832. doi: 10.3389/fpubh.2023.1139832 (PMC10043440; doi:10.3389/fpubh.2023.1139832)
Supplement: Supplementary file 5 [file Table_5.DOCX]

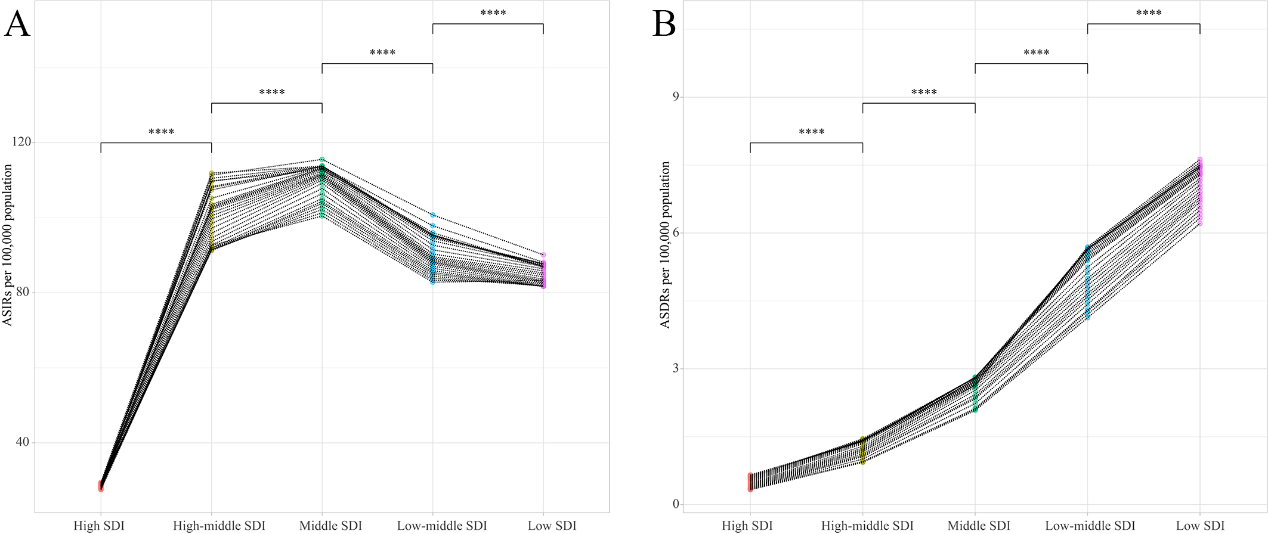
Figure S1. Line plots of ASIRs (A) and ASDRs (B) over 30 years for five SDI regions. Each dot represents the value of ASIRs or ASDRs for each year. Dots of the same year are connected by line segments. Paired t test was performed for adjacent SDI grades. ∗∗∗∗P < 0.0001.


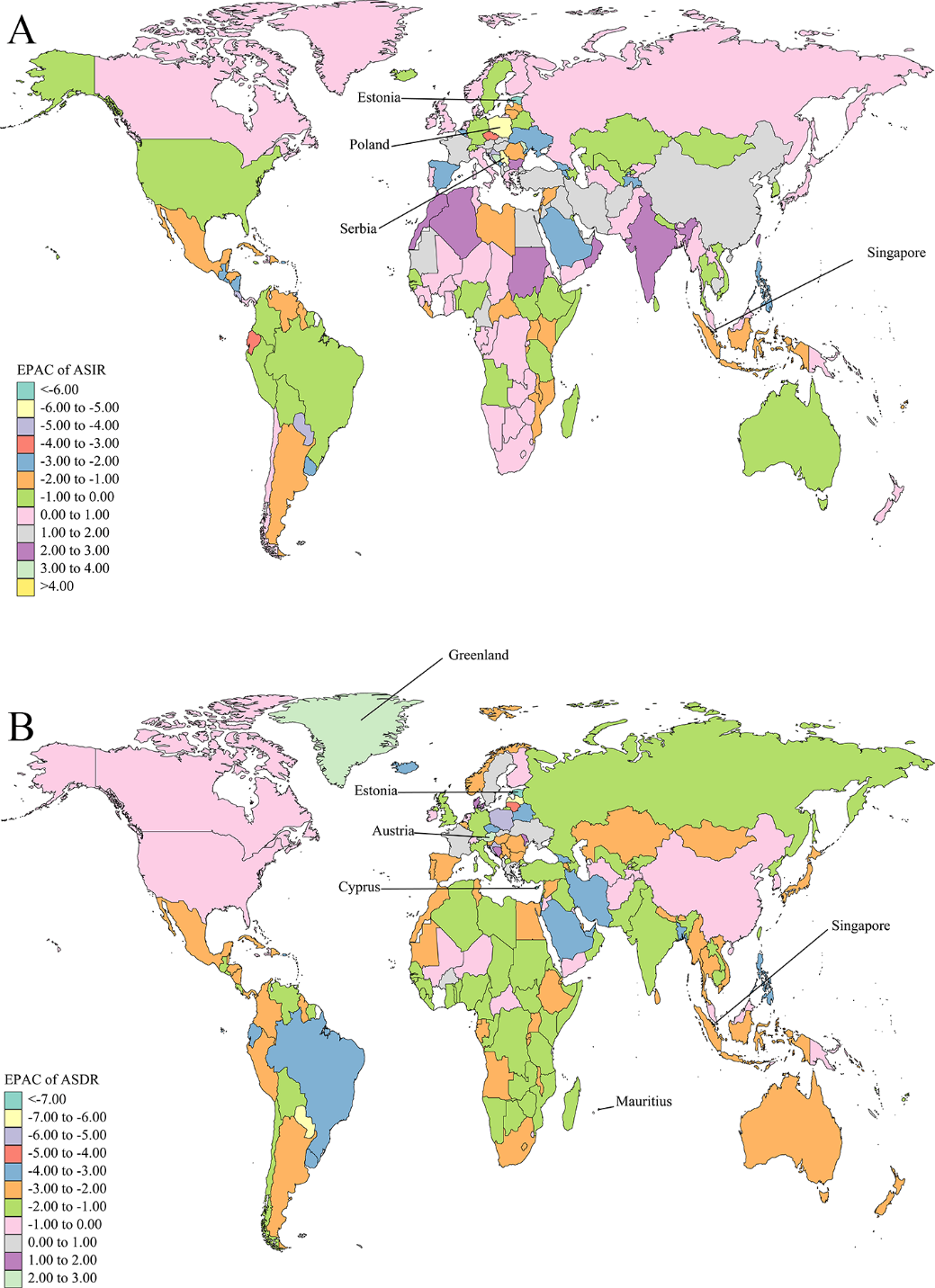
Figure S2. Global Trends in the Deaths of NSNIs in 204 Countries and Territories in the past 10 years. Estimated annual percentage changes (EAPCs) of ASIRs (A) and ASDRs (B) of NSNIs from 2010 to 2019 were shown.
